# Clinimetric properties of a novel feedback device for assessing gait parameters in stroke survivors

**DOI:** 10.1186/1743-0003-11-30

**Published:** 2014-03-05

**Authors:** Michiel Punt, Belinda van Alphen, Ingrid G van de Port, Jaap H van Dieën, Kathleen Michael, Jacqueline Outermans, Harriet Wittink

**Affiliations:** 1 Research group Lifestyle and Health, Utrecht University of Applied Sciences, Utrecht, The Netherlands; 2 Move Research Institute Amsterdam, Faculty of Human Movement Sciences, VU University Amsterdam, Amsterdam, The Netherlands; 3 King Abdulaziz University, Jeddah, Saudi Arabia; 4 University of Maryland, Baltimore, USA; 5 Revant Rehabilitation CentreBreda, Breda, The Netherlands

**Keywords:** Stroke, Ambulation, Step activity, Accelerometry, Feedback

## Abstract

**Introduction:**

Community-dwelling stroke survivors tend to become less physically active over time. There is no ‘gold standard’ to measure walking activity in this population. Assessment of walking activity generally involves subjective or observer-rated instruments. Objective measuring with an activity monitor, however, gives more insight into actual walking activity. Although several activity monitors have been used in stroke patients, none of these include feedback about the actual walking activity. FESTA (FEedback to Stimulate Activity) determines number of steps, number of walking bouts, covered distance and ambulatory activity profiles over time and also provides feedback about the walking activity to the user and the therapist.

**Aim:**

To examine the criterion validity and test-retest-reliability of the FESTA as a measure of walking activity in patients with chronic stroke. To target the properties of the measurement device itself and thus exclude effects of behavioral variability as much as possible evaluation was performed in standardized activities.

**Methods:**

Community-dwelling individuals with chronic stroke were tested twice with a test-retest interval varying from two days to two weeks. They performed a six-minute walk test and a standardized treadmill test at different speeds on both testing days. Walking activity was expressed in gait parameters: steps, mean-step-length and walking distance. Output data of the FESTA on the treadmill was compared with video analysis as the criterion measurement. Intraclass Correlations Coefficients (ICCs) and Mean Relative Root Squared Error (MRRSE) were calculated.

**Results:**

Thirty-three patients were tested to determine criterion validity, 27 patients of this group were tested twice for test-retest reliability. ICC values for validity and reliability were high, ranging from .841 to .972.

**Conclusion:**

This study demonstrated good criterion validity and test-retest-reliability of FESTA for measuring specific gait parameters in chronic stroke patients. FESTA is a valid and reliable tool for capturing walking activity measurements in stroke, and has applicability to both clinical practice and research.

## Introduction

In many Western nations, stroke is a leading cause of death and serious long-term disability [1]. A frequent consequence of stroke is unilateral loss or limitation of muscle function, leading to a loss of mobility, movement and functional ability [2,3]. Van de Port et al. (2006) showed that a substantial proportion of community-dwelling stroke survivors becomes less physically active over time [4]. Post-stroke physical inactivity may produce physical deconditioning, and as a consequence a decline in function [5]. A decline in function reduces participation in the community and quality of life [6] and decreases independence of the stroke survivor [5]. Furthermore, physical inactivity increases the risk of developing co-morbidities and having a recurrent stroke [5]. Accurate measurement of real life walking activity could be beneficial in tailoring rehabilitation. Using actual performance data and providing feedback might support self-management strategies to prevent physical and functional decline and subsequent consequences.

Currently assessment of walking activity generally involves subjective or observer-rated instruments [7]. These instruments have disadvantages such as the risk of recall bias, social desirability of answers, and poor generalisation [7]. Objective assessment of the number of steps can be done with pedometers. Roos et.al. (2012) demonstrated the disadvantage of measuring only the total number of steps taken [8]. They found differences in walking bouts and walking time between older adults and stroke survivors and that it varied based on functional ability. This relevant variation could not have been identified when measuring only steps per day [8]. Measuring gait parameters with accelerometers overcomes the limitation of measuring only the number of steps. To measure gait parameters by accelerometry in this population specific algorithms are required since stroke survivors are slow walkers [9] and accuracy of detecting steps decreases when gait speed and step frequency decrease [10]. To date, several motion sensors have been used [11,12], such as the accelerometer based StepWatch Activity Monitor (SAM) which has good validity in measuring gait parameters in stroke survivors. However, current devices are not capable of providing feedback to the stroke survivor about their walking activity. Providing feedback about their walking activity might prevent physical inactivity, and as a consequence a decline in function [5]. To monitor walking and to investigate potential beneficial effects of feedback in stroke survivors we developed FESTA.

FESTA (FEedback to STimulate Activity) is a telemetric system that includes a tri-axial piezo capacitive accelerometer which can be coupled to a docking station. The docking station is capable of; calculating gait parameters, evaluating whether the amount of walking activity during the day was sufficient according to the goal set by the physical therapist, providing the feedback to a screen visible for the stroke patient, sending an email towards the physical therapist with the calculated gait parameters and recharging the battery of the accelerometer to continue monitoring the next day.

As measuring gait parameters is more challenging in stroke survivors, the first step in this developing process was to examine the criterion validity and test-retest reliability of FESTA at gait parameter recognition in chronic stroke survivors using a stroke specific developed algorithm. We examined gait parameters; number of steps, mean-step-length with a standardized treadmill test and walking distance with an over ground 6 minute walk test. Furthermore FESTA calculates walking time and walking bouts as a derivative from steps [13].

## Methods

### Participants

A convenience sample of community-dwelling, chronic stroke survivors was recruited from ten private physical therapy practices, the daycare center of ‘Zorgspectrum’ and the patients’ association ‘Samen verder’ in the Netherlands and the University of Maryland in the United States of America. Stroke was defined according to the World Health Organization definition. Participants were able to walk independently without physical assistance (Functional Ambulation Categories score ≥ 3) [14] and were at least three months post stroke.Participants were excluded if they had severe cognitive disorders (Mini-Mental State Examination <24) [15], severe communicative disorders (Utrechts Communicatie Onderzoek <4) [16] or acute disorders impairing gait. All participants gave written informed consent prior to participation in the study. The research protocol and all informational material were approved by the Medical Ethical Committee (MEC) of the University Medical Center Utrecht and the Institutional Review Board of the University of Maryland, Baltimore. Treatment of the participants was according to the Helsinki declaration [17].

### Equipment & experimental protocol

#### Procedure

Participants were tested twice with a test-retest interval of a minimum of two days and a maximum of two weeks using the six-minute walk test (6MWT) and a standardized treadmill test. At baseline, inclusion measurements and collection of personal and anthropometric data were performed prior to the physical tests.

#### FESTA monitor

During both tests the FESTA was worn around the back site of the waist, between the posterior superior iliac spines. The FESTA contains one tri-axial, piezo-capacitive accelerometer (70*80*25 mm, 150 grams, range ± 2.5 g). Based on sensor alignment, acceleration signals were identified as anterior-posterior (AP), medio-lateral (ML) and vertical (VT). Output is in mV, a change of 1 mV corresponded to a change of 0.08 m/s^2^ (resolution)). Acceleration signals were digitally stored on a memory card with a sampling rate of 25 samples/s.

### 6MWT

The 6MWT was performed to assess overground walking distance. The 6MWT was performed according to the American Thoracic Society Guidelines [18]. Walked distance was determined by counting the number of walked laps (20 meters) and adding any final fraction of laps, measured by a measuring wheel. Results were used to calculate the comfortable walking speeds for the treadmill test (CWT) and to assess the overground walking distance validity and reliability of the FESTA.

#### Standardized treadmill test

Gait parameters, number of steps and mean-step-length were determined using a standardized treadmill test. Because accuracy of the gait parameter; steps recognition depends on gait speed and gait speed may vary during a day and is low in this population [9] we executed a treadmill test at three different gait speeds within each subject. Gait speeds were established at 15% below, equally to and 15% above comfortable gait speed. Each speed condition lasted for two minutes. The mean walking speed measured by the 6MWT −10% was used to define the comfortable walking speed. Fingertip handrail support was allowed during testing. The treadmills (En Mill treadmill, Enraf Nonius, the Netherlands and Gait Trainer 3™, Biodex, USA) were calibrated prior to the study. A camera was placed 1.2 meter behind the treadmill (Panasonic type HC-V70, 50 samples/s).

### Data processing and algorithms

From every block of two minutes at different speeds, only the last 90 seconds were analysed. The researcher counted the number of steps during these blocks of 90 seconds from the video afterwards and was blinded from the results of FESTA. Distances from the treadmill test were determined by using the treadmill speed and the testing time of the treadmill test. The mean-step-length was derived from the distance walked and divided by the steps taken by both legs.

From the same blocks of 90 seconds, the gait parameters (number of steps and mean-step-length) from FESTA were analysed using Matlab (Matlab 7.10.1, The MathWorks Inc, USA).For the step detection we used spectral analysis derived from the AP acceleration signal. Taking the individual variety of the distance-acceleration relationship into account, we used an individual calibration procedure for distance measures to determine the acceleration-distance relation [19]. Firstly we calculated the root mean square of the AP acceleration signal, secondly conducted a linear fit (first order polynomial) between the different gait speeds and the different root mean square values, thirdly we used the polynomial function to predict the walking speed and subsequently walking distance in the treadmill test and 6MWT.This distance prediction derived from a single acceleration signal and the individual calibration procedure is described by Schutz et al. (2002) [19] in more detail.

To assess the validity and reliability of FESTA, we compared the gait parameters derived from FESTA with the gold standard (video analysis). Comparisons for the gait parameters number of steps and mean-step-length were performed by using the data from the treadmill test. This procedure is consistent with procedures from similar validation studies [20-23] and video analysis seems to be the most appropriate criterion standard for the assessment of physical activity [23]. The comparison of the gait parameter walking distance was performed using the data derived from the 6MWT.

### Statistics

Descriptive statistics were performed for all variables and normality was assessed by visual inspection of histograms and quantile-quantile plots. An ICC_3,1_ of ≥ .75 was defined as high as suggested by Burdock et al.(1963) [24]. All calculations were performed using SPSS (IBM Software, SPSS Statistics 20, USA) or Matlab (Matlab 7.10.1, The MathWorks Inc, USA).

### Validity

To assess the level of agreement between FESTA and the gold standard, and thus the criterion validity, single measures intraclass correlation coefficients_agreement_ (ICC_3,1_, Two-way mixed model) were calculated for the different gait parameters; number of steps and mean-step-length obtained from the treadmill test and over ground walked distance in the 6MWT. Furthermore the Mean Relative Root Square Error (MRRSE) was calculated for each parameter. The MRRSE is a measure of the differences between the values of FESTA and the observed values, relative to the unit of measurement (see Formula). The MRRSE gives an indication of the mean error of FESTA per step or number of steps as a percentage of the measurement unit.

$$\mathrm{MRRSE}=\frac{\sqrt{\sum_{i=1}^{n}(\mathrm{Xobs},i-X\;\mathrm{model},i}){}^{2}}{{\bar{X}}_{\mathrm{obs}}}*100$$

${\bar{X}}_{\mathrm{obs}}$*= mean of the observed values, criterion measurement, video analysis*

X_*mo del*,*i*_*= values obtained by FESTA*

### Reliability

Single measures intraclass correlation coefficients_consistency_ (ICC_3,1_, Two-way mixed model) was calculated to analyse the test-retest reliability of FESTA. Additionally, the Minimal Detectable Change (MDC_95_) was calculated from the Standard Error of Measurement (SEM) as MDC_95_ = [1.96*SEM_consistency_ *√2] and SEM = [sd * $\sqrt{\left[1-r\right]\;}$, where *r* is the test-retest reliability coefficient ICC_agreement 3,1_ and sd is the standard deviation of the scores at the first test occasion (T0). The SEM is multiplied by 1.96 to determine the 95% confidence interval and multiplied by the square root of 2 to account for the additional error associated with repeated measurements [25]. The MDC_95_ is the minimal amount of change that must be observed before the change can be considered to exceed the variation and measurement error at the 95% confidence level.

## Results

A total of 33 participants (17 men and 16 women) were tested and their data were used to determine the criterion validity of FESTA. Twenty-seven participants were tested twice. The other six participants did not perform a second test, due to motivational problems to perform a second test or being unable to perform a second test within the set time limit of two weeks after the first test. The mean age of the 33 participants was 61.8 ± 8.8 years, time since stroke was 5.6 years ± 3.8 years and the functional ambulation category (FAC) scores ranged from 3 to 5 (mean 4.4 ± 0.7). The average distance walked in the 6MWT was 317.3 meters, which is 0.88 m/s, ranging from 36 to 580 meters. For the treadmill testing, the different walking speeds varied from 0.08 to 1.5 m/s.

### Validity

For number of steps and mean-step-length at the three different gait speeds, ICC_agreement__3,1_ varied between 0.841 and 0.971 (p ≤ 0.001 for all values). Mean Relative Root Squared Errors (MRRSE) ranged between 3.4 and 9.1%. All agreement parameters are presented in Table 1.

**Table 1:** Validity results FESTA

**Speed**	**Parameter**	**Video analysis**	**FESTA**	**MRRSE (%)**	**ICC**
Speed 1 = CWT - 15%		*Mean ± SD*	*Mean ± SD*		
Mean ± SD: 2.4 ± 1.1 km/h	Step count (steps)	129 ± 25	135 ± 21	5.8	.841
Range: 0.3 - 4.4 km/h	Mean step length (m)	0.45 ± 0.14	0.43 ± 0.16	9.1	.910
Speed 2 = CWT					
Mean ± SD: 2.8 ± 1.2 km/h	Step count (steps)	138 ± 27	141 ± 23	3.5	.964
Range: 0.4 - 5.2 km/h	Mean step length (m)	0.50 ± 0.15	0.48 ± 0.16	6.2	.964
Speed 3 = CWT + 15%					
Mean ± SD: 3.2 ± 1.4 km/h	Step count (steps)	145 ± 28	146 ± 25	3.4	.964
Range: 0.5 - 5.6 km/h	Mean step length (m)	0.54 ± 0.17	0.52 ± 0.18	5.3	.971

MRRSE = Mean Relative Root Squared Error; percentage mean absolute deviation, ICC = Intraclass Correlation Coefficient, CWT = Comfortable Walking Speed for Treadmill.

**Figure 1:**
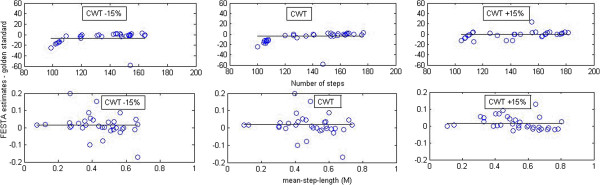
**FESTA estimates of steps minus golden standard (top panel).** FESTA estimates of mean-step-length minus golden standard (bottom panel). At 15% below comfortable walking speed (CWT −15%) equal to (CWT) and 15% above.

Figure 1 illustrates the differences between the gold standard and FESTA for the gait parameters steps and mean-step-length, with the difference in steps (top panel) and mean-step-length (bottom panel).

**Figure 2:**
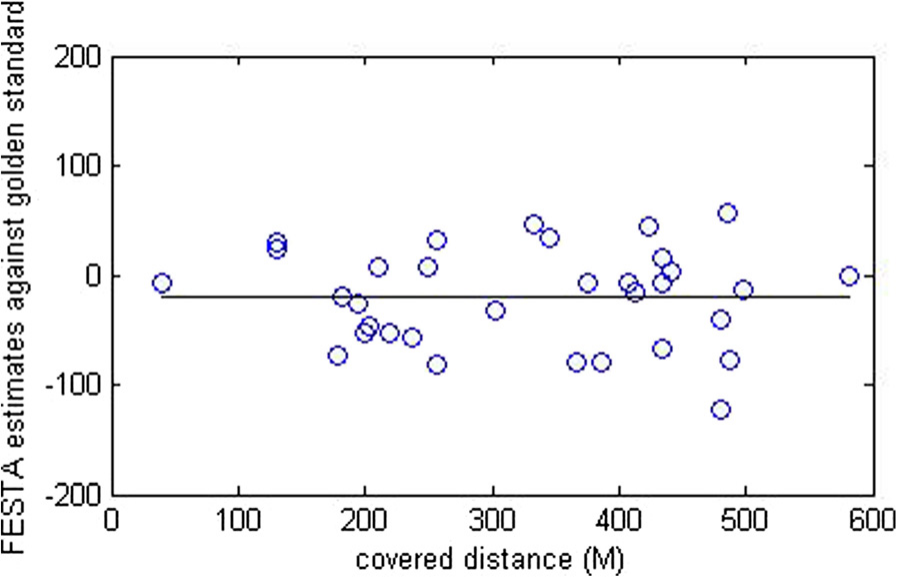
FESTA estimates of covered distance (M) minus golden standard at the 6MWT.

Criterion validity for overground walking distance during the 6MWT is presented in Table 2. The difference between measured and estimated over ground walking distance in meters averaged −20.1 meters see Figure 2.

**Table 2:** Validity overground walking distance

	**6MWT (m) measured**	**6MWT (m) FESTA**	**MRRSE (%)**	**ICC**
*Mean ± SD*	317.3 ± 134.7	337.4 ± 136.3	12.1	.937
*Range*	36.0 -580.0	44-581.5		

6MWT = Six Minute Walk Test, MMRSE = Mean Relative Root Squared Error; percentage mean absolute deviation, ICC = Intraclass Correlation Coefficient.

### Reliability

Table 3 presents the test-retest reliability for the gait parameters steps and mean-step-length, including ICC values and MDC. ICC_consistency 3,1_ scores ranged from 0.876 to 0.972 and were all significant at p ≤ 0.001.

**Table 3:** Test-retest reliability of gait parameters obtained by FESTA

**Speed**	**Parameter**	**T0 (*Mean ± SD)***	**T1 (*Mean ± SD)***	**ICC**	**MDC**_**95**_
Speed 1 = CWT - 15%	Step count (steps)	136 ± 21.0	135 ± 20.4	.938	14.3
	Mean step length (m)	0.44 ± 0.15	0.42 ± 0.14	.876	0.14
Speed 2 = CWT	Step count (steps)	141 ± 22.5	140 ± 22.6	.949	14.1
	Mean step length (m)	0.48 ± 0.14	0.48 ± 0.15	.942	0.10
Speed 3 = CWT + 15%	Step count (steps)	145 ± 23.9	144. ± 25.6	.972	11.4
	Mean step length (m)	0.52 ± 0.16	0.53 ± 0.16	.944	0.10

CWT = Comfortable Walking speed for Treadmill, ICC = Intraclass Correlation Coefficient, MDC = Minimal Detectable Change.

**Figure 3:**
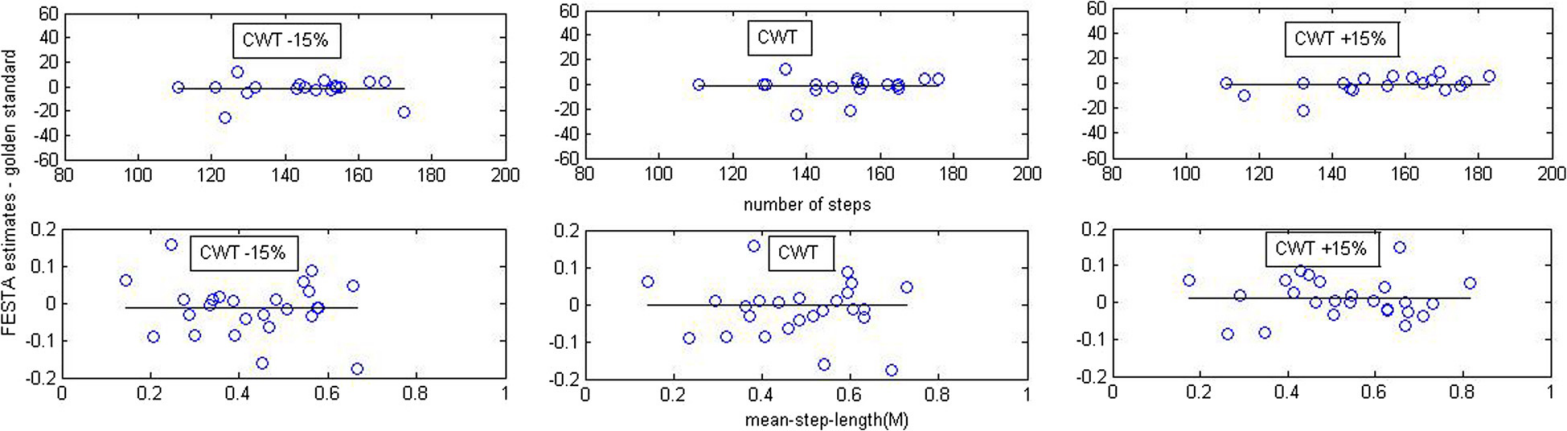
**FESTA estimates, difference between steps at the first and second test occasion (top panel).** FESTA estimates, difference between mean-step-length at the first and second test occasion (bottom panel).

Figure 3 illustrates the differences between the first and second test occasion, reliability of the gait parameters; steps (top panel) and mean-step-length (bottom panel).

**Figure 4:**
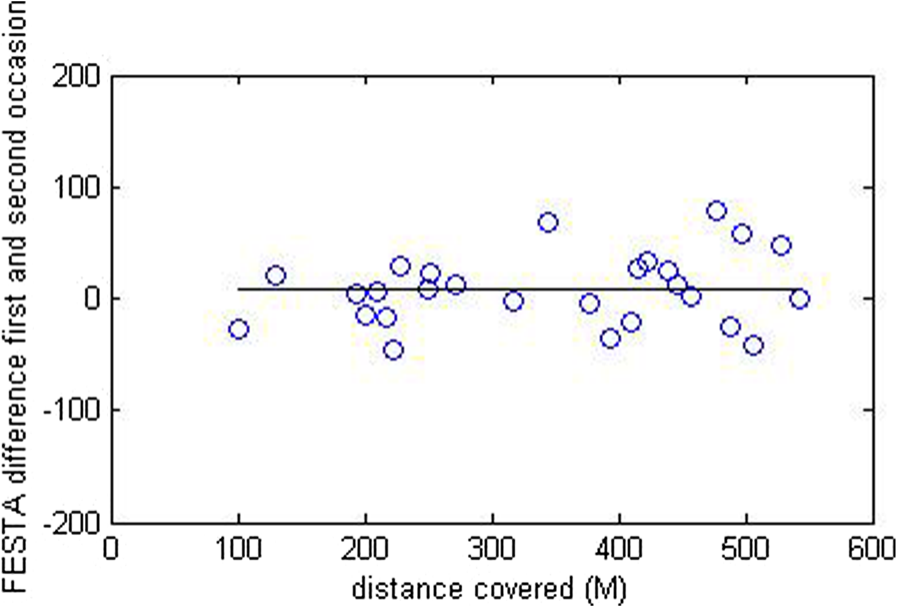
FESTA estimates difference in covered walking distance (M) between the first and second test occasion.

Test-retest reliability for over ground distance covered during the 6MWT for ICC_agreement 3,1_ ,was .97. Mean difference in meters was 8.1 meter, see Figure 4.

## Discussion

The objective of this study was to examine the criterion validity and test-retest reliability of the novel telemetric system, FESTA, in measuring walking activity in stroke survivors. To this end, we tested gait parameters; steps, mean-step-length and walking distance in chronic stroke survivors. Results of criterion validity and test-retest reliability indicate good validity and reliability as all ICC values were between .841 and .972. These results are similar to the most commonly used accelerometer in the stroke population [10,12,26]. Moreover, the results present higher accuracy in comparison to algorithms not specifically developed for the stroke population [26].

No clear trend can be seen between ICC values and MRRSE and the three gait speed conditions. This indicates that the validity of the FESTA is not affected by gait speed. Although the latter finding demonstrates the possible robustness of the FESTA for real-life use, we have to take into account that gait parameters differ for treadmill walking and over ground walking [27]. When walking on a treadmill, the gait patterns of chronic stroke survivors are more symmetrical and stable compared to overground walking. Furthermore in real-life gait speed may vary during a day and even within a walking bout. Therefore the gait parameters steps and mean-step-length have to be interpreted with caution since these parameters were only tested on the treadmill and might not be generalizable to walking overground. Further research is needed to determine these outcomes in overground walking. Another limitation of the study was the test-retest reliability design. Although all conditions were similar in the first and second test occasion and subjects were stable, subjects did perform slightly differently in the first and second test. For example, subjects took slightly fewer steps in the treadmill test and walked a few meters further in the second 6MWT compared to the first test occasion. This affected the reliability results of FESTA.

For specific measurement devices, measurement errors should be smaller than the Minimal Clinically Important Difference (MCID) to detect a valuable clinical effect for individuals. For the treadmill tests, no MCIDs have been defined. The MDC_95_ score of the FESTA for overground walked distance at the 6MWT was 62.2 meters. Unfortunately the interpretation of this value remains unclear as the MDC of FESTA depends on de MDC of the 6MWT since the MDC of FESTA is derived during the 6MWT. The MDC of the 6MWT for this population ranges from 29 m [28] towards 54.1 m [29].

### Statistical considerations

Previous studies with a similar design and aim as we had [11,12,30,31] expressed accuracy performance in ICC values and Limits of Agreement. In this study, we added a new measure for validity; the Mean Relative Root Squared Error (MRRSE). It is known that ICC values are strongly influenced by the magnitude of the variance within the study sample. Furthermore, other than the name ICC_agreement_ suggests, the ratio of variances is calculated, rather than the absolute agreement score [32]. When taking a closer look at the ICC formula, it is clear that a large variance in subject scores, as is the case in this study, will lead to a higher ICC [32]. Studies with different variances in their study populations can therefore not be compared directly. To get a better insight in the true agreement between the output of the FESTA and the ‘gold standard’, and to eliminate the effect of the high variance in our study population, we calculated the MRRSE for each gait parameter. The MRRSE represents the mean absolute percentage difference between the two measurement devices, expressed in the percentage of unit of the parameter. This score is easy to use in daily practice, easy to interpret and not dependent of variance between patients. Therefore, we hereby suggest using the MRRSE in future research, as it provides a more direct comparison between studies and between measurement devices.

### FESTA

FESTA (FEedback to STimulate Activity) is a newly developed telemetric system and validity and reliability were shown to be good in the present analysis. It is designed to monitor and stimulate stroke survivors with respect to their daily walking activity. The physical therapist is able to interact by setting walking activity goals based on walking time and walking bouts. FESTA has several advantages over other methods for assessing walking activity; it can measure different gait parameters such as number of steps, mean-step-length, distance and as derivatives walking time and the number of walking bouts [13], whereas a step-counter can only determine the number of steps. Roos et. al. (2012) clearly stated that steps alone is not sufficient to characterize physical inactivity in stroke survivors [8]. Due to the docking station FESTA is not limited by battery life and data capacity. Therefore it is able to monitor for a long time period without recharging or removing data. Furthermore FESTA provides the researcher, physical therapist and stroke survivor with real-life walking activity information. Future research will involve studying the effect of giving feedback using this device. The aim will be to increase walking activity by providing feedback to the user and providing information of actual walking activity and the daily pattern of walking activity to the physical therapist. Using FESTA provides new possibilities to measure walking activity of chronic stroke survivors in a valid and reliable way and thereby offers a variety of perspectives for research and treatment in this population.

## Conclusion

Based on ICC values and MRRSE, this study demonstrated good criterion validity and test-retest reliability of the telemetric system FESTA for measuring gait parameters in chronic stroke survivors. FESTA provides the possibility to measure gait parameters in a valid and reliable manner and can be used, in both clinical practice and academic research.

## Abbreviations

FESTA: FEedback to STimulate Activity; ICC: Intra class correlation; SEM: Standard error of measurement; MDC: Minimal detectable change; MRRSE: Mean relative root squared error; 6MWT: Six minute walk test; CWT: Comfortable walking speed for treadmill testing.

## Competing interests

The authors state that there was no conflict of interests with any financial or personal relationships or organizations that could influence the research results.

## Authors’ contribution

MP has made substantial contribution in the experimental design, algorithm development, interpretation and analysis of data and drafting the manuscript. BA has made substantial contribution in analyzing the data and drafting the manuscript. IP has made substantial contribution in facilitating the research project and revising the manuscript. JD has made substantial contribution in revising the manuscript. KM has made substantial contribution in acquisition in subjects and revising the manuscript. JO has made substantial contribution in acquisition in subjects and revising the manuscript. HW has made substantial contribution in facilitating the research project, drafting and revising the manuscript. All authors read and approved the final manuscript.

## References

[B1] English C Hillier S Circuit class therapy for improving mobility after stroke: a systematic review J Rehabil Med Off J UEMS Eur Board Phys Rehabil Med 2011 43 Suppl 7 565 571 10.2340/16501977-0824 21584485

[B2] Indredavik B Rohweder G Naalsund E Lydersen S Medical complications in a comprehensive stroke unit and an early supported discharge service Stroke 2008 39 Suppl 2 414 420 18096834 10.1161/STROKEAHA.107.489294

[B3] Langhorne P Stott DJ Robertson L MacDonald J Jones L McAlpine C Dick F Taylor GS Murray G Medical complications after stroke: a multicenter study Stroke 2000 31 Suppl 6 1223 1229 10835436 10.1161/01.str.31.6.1223

[B4] van de Port IGL Kwakkel G van Wijk I Lindeman E Susceptibility to deterioration of mobility long-term after stroke: a prospective cohort study Stroke 2006 37 Suppl 1 167 171 16322486 10.1161/01.STR.0000195180.69904.f2

[B5] Brazzelli M Saunders DH Greig CA Mead GE Physical fitness training for stroke patients Cochrane Database Syst Rev(online) 2011 11 1 109 10.1002/14651858.CD003316.pub4 22071806

[B6] Pound P Gompertz P Ebrahim S A patient-centred study of the consequences of stroke Clin Rehabil 1998 12 Suppl 4 338 347 9744669 10.1191/026921598677661555

[B7] Pearson OR Busse ME van Deursen RWM Wiles CM Quantification of walking mobility in neurological disorders QJM 2004 97 Suppl 8 463 475 15256604 10.1093/qjmed/hch084

[B8] Roos MA Rudolph KS Reisman DS The structure of walking activity in people after stroke compared with older adults without disability: a cross-sectional study Phys Ther 2012 92 Suppl 9 1141 1147 22677293 10.2522/ptj.20120034 PMC3432950

[B9] Goldie PA Matyas TA Evans OM Deficit and change in gait velocity during rehabilitation after stroke Arch Phys Med Rehabil 1996 77 1074 1082 10.1016/S0003-9993(96)90072-6 8857890

[B10] Taraldsen K Askim T Sletvold O Einarsen EK Bjåstad KG Indredavik B Helbostad JL Evaluation of a body-worn sensor system to measure physical activity in older people with impaired function Phys Ther 2011 91 Suppl 2 277 285 21212377 10.2522/ptj.20100159

[B11] Saremi K Marehbian J Yan X Regnaux JP Elashoff R Bussel B Dobkin BH Reliability and validity of bilateral thigh and foot accelerometry measures of walking in healthy and hemiparetic subjects Neurorehabil Neural Repair 2006 20 Suppl 2 297 305 16679506 10.1177/1545968306287171

[B12] Mudge S Stott NS Walt SE Criterion validity of the StepWatch Activity Monitor as a measure of walking activity in patients after stroke Arch Phys Med Rehabil 2007 88 Suppl 12 1710 1715 18047890 10.1016/j.apmr.2007.07.039

[B13] Orendurff MS Schoen JA Bernatz GC Segal AD Klute GK How humans walk: bout duration, steps per bout, and rest duration J Rehabil Res Dev 2008 45 Suppl 7 1077 1090 19165696 10.1682/jrrd.2007.11.0197

[B14] Holden MK Magliozzi MR Nathan J Piehl-Baker L Clinical gait assessment in the neurologically impaired: reliability and meaningfulness Phys Ther 1984 64 Suppl 1 35 40 6691052 10.1093/ptj/64.1.35

[B15] Folstein FSE McHugh MF “Mini-mental state". a practical method for grading the cognitive state of patients for the clinician J Psychiatr Res 1975 12 Suppl 3 189 1202204 10.1016/0022-3956(75)90026-6

[B16] Back PMWMFZ van der Velden H Schepers VPM Visser-Meily JMA De Spontane Communicatieschaal van het Utrechts Communicatie Onderzoek: Een valide screener van communicatieve vaardigheden Revalidata 2006 28 Suppl 133 p5

[B17] World Medical Association, 2002 Declaration of Helsinki Ethical principles for medical research involving human subjects J Postgrad Med 2002 48 suppl 3 206 208 12432198

[B18] ATS Committee on Proficiency Standards for Clinical Pulmonary Function Laboratories ATS statement: guidelines for the six-minute walk test Am J Respir Crit Care Med 2002 166 Suppl1 111 117 12091180 10.1164/ajrccm.166.1.at1102

[B19] Schutz Y Weinsier S Terrier P Durrer D A new accelerometric method to assess the daily walking practice Int J Obes 2002 26 111 118 10.1038/sj.ijo.0801856 11791155

[B20] Terrier P Aminian K Schutz Y Can accelerometry accurately predict the energy cost of uphill/downhill walking? Ergonomics 2001 44 suppl 1 48 62 11214898 10.1080/00140130118289

[B21] Houdijk H Appelman FM Van Velzen JM Van der Woude LH Van Bennekom CA Validity of DynaPort GaitMonitor for assessment of spatiotemporal parameters in amputee gait J Rehabil Res Dev 2008 45 suppl 9 1335 1342 19319757

[B22] Bussmann JB Martens WL Tulen JH Schasfoort FC van den Berg-Emons HJ Stam HJ Measuring daily behavior using ambulatory accelerometry: the activity monitor Behav Res Methods Instrum Comput 2001 33 suppl 3 349 56 11591066 10.3758/bf03195388

[B23] Sirard JR Pate RR Physical activity assessment in children and adolescents Sports Med 2001 31 suppl 6 439 454 11394563 10.2165/00007256-200131060-00004

[B24] Burdock EI Fleiss JL Hardesty AS A new view of inter-observer agreement Pers Psychol 1963 16 suppl 4 373 384

[B25] Haley SM Fragala-Pinkham MA Interpreting change scores of tests and measures used in physical therapy Phys Ther 2006 86 suppl 5 735 743 16649896

[B26] Fulk GD Combs SA Danks KA Nirider CD Raja B Reisman DS Accuracy of two activity monitors in detecting steps in people with stroke and traumatic brain injury Phys Ther 2014 94 suppl xxx 1 34 10.2522/ptj.20120525 24052577

[B27] Harris-Love ML Forrester LW Macko RF Silver KH Smith GV Hemiparetic gait parameters in overground versus treadmill walking Neurorehabil Neural Repair 2001 15 suppl 2 105 112 11811252 10.1177/154596830101500204

[B28] Fulk GD Echternach JL Nof L O’Sullivan S Clinometric properties of the six-minute walk test in individuals undergoing rehabilitation poststroke Physiother Theory Pract 2008 24 suppl 3 195 204 18569856 10.1080/09593980701588284

[B29] Liu J Drutz C Kumar R McVicar L Weinberger R Brooks D Salbach NM Use of the six-minute walk test poststroke: is there a practice effect? Arch Phys Med Rehabil 2008 89 Suppl 9 1686 1692 18760152 10.1016/j.apmr.2008.02.026

[B30] Edbrooke L Lythgo N Goldsworthy U Denehy L Can an accelerometer-based monitor be used to accurately assess physical activity in a population of survivors of critical illness? Global Journal of Health Science 2012 4 suppl 3 98 107 22980236 10.5539/gjhs.v4n3p98 PMC4776921

[B31] Hale LA Pal J Becker I Measuring free-living physical activity in adults with and without neurologic dysfunction with a triaxial accelerometer Arch Phys Med Rehabil 2008 89 suppl 9 1765 1771 18760161 10.1016/j.apmr.2008.02.027

[B32] McGraw KO Wong SP Forming inferences about some intraclass correlation coefficients Psychol Methods 1996 1 suppl 1 30 46

